# Preparing Geriatrics-Trained Physicians to Discuss Medical Cannabis with their Older Adult Patients

**DOI:** 10.1093/geroni/igaf122.4179

**Published:** 2025-12-31

**Authors:** Alison Moore, Ali Punsalan, Krish Jagasia, Julie Bobitt, Jeremy Hirst, Michelle Sexton, Kathryn Winters, Annie Nguyen

**Affiliations:** University of California San Diego, San Diego, California, United States; UC San Diego, La Jolla, California, United States; UC San Diego, La Jolla, California, United States; University of Illinois at Chicago, Chicago, Illinois, United States; UC San Diego, La Jolla, California, United States; UC San DIego, La Jolla, California, United States; UC San Diego, La Jolla, California, United States; UC San Diego, La Jolla, California, United States

## Abstract

Older adults are increasingly using cannabis for health conditions (e.g., pain, sleep disorders), often without consulting a healthcare provider. Many providers also feel unprepared to discuss cannabis use with patients. This educational intervention aimed to raise geriatrics-trained physician awareness and promote compassionate dialogue about cannabis use with older adults. Thirty-one geriatrics and geriatric psychiatry physicians and fellows attended a synchronous, online, didactic series on cannabis divided into four, 30-minute modules on: 1) epidemiology of use among older adults, 2) history of legalization and stigma associated with use, 3) medical evidence and patient advising, and 4) having compassionate dialogue related to use. Pre- and post-intervention data were collected (n = 22 pre-test, n = 13 post-test). Wilcoxon signed-rank tests were performed to assess changes. Statistically significant (p-value’s<.05) increases were seen in the percentage of physicians who reported being: moderately to extremely knowledgeable about medical cannabis (18.2% to 71.4%), somewhat to very comfortable initiating conversations about cannabis (22.7% to 92.9%), very to extremely confident assessing the risks (4.5% to 28.6%), benefits (0% to 14.3%), and safety of cannabis (0% to 21.4%), and very to extremely confident providing advice about cannabis (0% to 14.2%). This brief training series was effective in raising physicians’ knowledge about and comfort with having conversations about medical cannabis with their older adult patients. Similar training can be easily implemented in pre- and post-doctoral settings. As more older adults turn to cannabis as a therapeutic alternative, physicians will need access to formal training to strengthen confidence providing advice.

